# Iron(ing) out parkinsonisms: The interplay of proteinopathy and ferroptosis in Parkinson's disease and tau-related parkinsonisms

**DOI:** 10.1016/j.redox.2024.103478

**Published:** 2024-12-19

**Authors:** Maria João da Costa Caiado, Amalia M. Dolga, Wilfred F.A. den Dunnen

**Affiliations:** a Graduate School of Medical Sciences (GSMS) and Research School of Behavioural and Cognitive Neurosciences (BCN), University of Groningen, 9713 GZ, Groningen, the Netherlands; b Department of Pathology and Medical Biology, University Medical Centre Groningen (UMCG), Hanzeplein 1, 9713 GZ, Groningen, the Netherlands; c Department of Molecular Pharmacology, Groningen Research Institute of Pharmacy (GRIP), University of Groningen, 9713 AV, Groningen, the Netherlands

**Keywords:** Iron homeostasis, Synucleinopathy, Tauopathy, Ferroptosis, Neurodegeneration, Mitochondria

## Abstract

Parkinsonian syndromes are characterised by similar motor-related symptomology resulting from dopaminergic neuron damage. While Parkinson's disease (PD) is the most prevalent parkinsonism, we also focus on two other variants, Progressive supranuclear palsy (PSP) and Corticobasal degeneration (CBD). Due to the clinical similarities of these parkinsonisms, and since definite diagnoses are only possible post-mortem, effective therapies and novel biomarkers of disease are scarce. Thus, we explore the current findings relating to the relationship of parkinsonism proteinopathy (α-synuclein in PD, and tau in PSP/CBD) paralleled to a specific form of cell death, ferroptosis. Ferroptosis is characterised by iron-induced lipid peroxidation and several markers of this pathway have been identified to control intracellular iron fluctuations. However, in parkinsonism, these mechanisms are thought to become dysfunctional. Although both proteinopathies have been linked to ferroptosis, much less is known about ferroptotic cell death and tau in the context of PSP/CBD. Interestingly, clinical trials targeting iron have recently shown conflicting results which begs to question the complexity of the ferroptotic pathway and alludes to the need for exploring other ferroptosis-related machinery as possible therapeutic targets. Overall, we address the literature gap in parkinsonism proteinopathy and ferroptosis, and its relevance to understanding disease pathophysiology and aetiology.

## Graphical abstract

**Figure:**
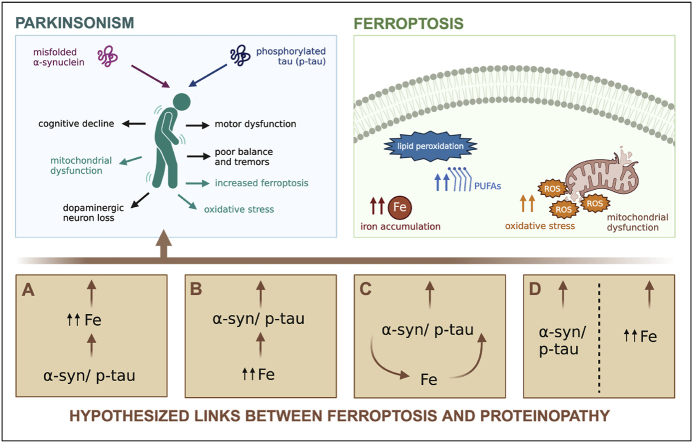

## 1 Introduction

### 1.1 Parkinsonian syndromes

Parkinsonian syndromes are described in (neurodegenerative) disorders characterised by similar clinical pictures of motor- and postural-related deficits, mainly resulting from damage of dopaminergic neurons in the substantia nigra (SN) [1]. While Parkinson's disease (PD) is the most common type of parkinsonism, affecting >1 % of the population aged 60 and over [2], and with an increasing trend reported in the Western world [3], other parkinsonian syndromes are less prevalent but also less recognised, often leading to a misdiagnosis or to a diagnostic delay [4]. Definite diagnoses are only possible post-mortem which, due to the clinical similarity of different parkinsonisms, further contributes to the ante-mortem diagnostic problem and thus, lack of adequate patient care [[5], [6], [7], [8], [9]]. Although correct diagnoses can help with choosing a more suitable course of treatment action, there is not a very large window of consideration given that therapies for atypical parkinsonisms are still scarce [1,10]. Additionally, diagnoses are necessary for patients and caregivers as it allows for insight into prognosis and clinical disease progression, as well as helping inform them how to manage life with a chronic illness [4]. Thus, this lack of disease identification contributes to poor patient care, and to a lack of translation of the research options to treat parkinsonian syndromes, as they are not easily identifiable nor recognisable. Consequently, clinical trials are consistently unsuccessful predictably due to inaccurate patient sampling during disease screening [1].

**Fig. 1:**
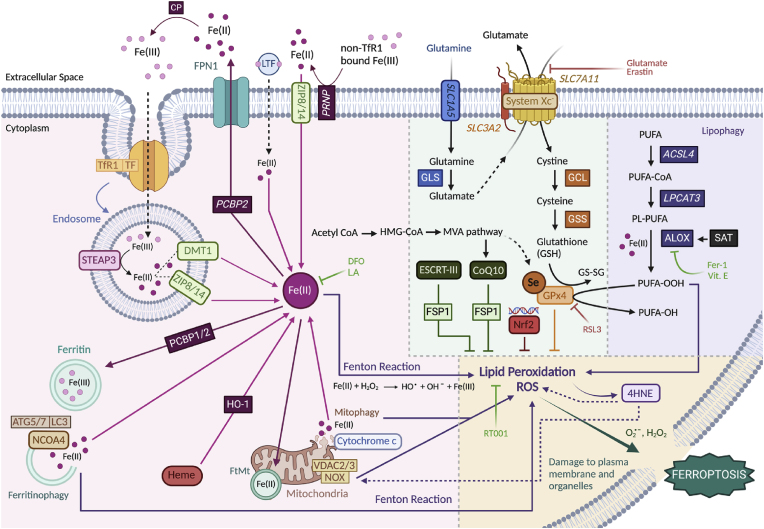
Ferroptosis-related pathways. Several pathways have been identified to be possibly contributing towards ferroptosis, either via excessive iron accumulation (represented in pink), toxic polyunsaturated fatty acid (PUFA) breakdown (purple), and ultimately, oxidative stress and cell membrane damage (yellow). Other mechanisms, such as glutathione metabolism, are also essential as they are thought to protect against ferroptosis (green), under healthy conditions. Exogenous ferroptosis inhibitors (green) and inducers (red), and their respective targets, are also represented. ACSL4, Acyl-CoA synthetase long chain family member 4; ATG5/7, Autophagy-related 5/7; CP, Ceruloplasmin; Fe(II), Ferrous iron; Fe(III), Ferric iron; GCL, Glutamate-cysteine ligase; GLS, Glutaminase(s); GS-SG, Glutathione disulphide; HMG-CoA, β-hydroxy β-methylglutaryl-CoA; LC3, Microtubule-associated protein 1A/1B-light chain 3; LPCAT3, Lysophosphatidylcholine acyltransferase 3; MVA, Mevalonate pathway; PCBP1/2, Poly(rC)-binding protein 1/2; PRNP, Major prion protein; PL-PUFA, Polyunsaturated fatty acid containing phospholipids; PUFA-OH, Polyunsaturated fatty acid alcohol; PUFA-OOH, Polyunsaturated fatty acid hydroxide; RSL3, Ras selective lethal 3; RT001, Di-deuterated ethyl linoleate; SAT, spermidine/spermine N1-acetyltransferase; Se, Selenocysteine; STEAP3, Six-Transmembrane Epithelial Antigen Of Prostate 3; SLC1A5, Solute carrier family 1 member 5 (neutral amino-acid transporter); SLC3A2, Solute carrier family 3 member 2 (Xc-subunit); SLC7A11, Solute carrier family 7 member 11 (Xc-subunit); VDAC2/3, Voltage dependent anion channel 2/3; Vit. E, Vitamin E; Xc-, Glutamate/cystine anti-porter system; ZIP8/14, solute carrier family 39 member 8/14 (divalent metal transporters).

The present review focuses primarily on PD, marked by alpha-synuclein (aS) pathology, and on two other specific forms of parkinsonian syndromes: Progressive supranuclear palsy (PSP) and Corticobasal degeneration (CBD), and their pathological hallmark, tau. In parallel, we try to bridge the literature gap in these disorders by exploring the current findings relating to the relationship of proteinopathy and ferroptosis, as well as discuss differences and similarities between ferroptosis-related markers in these parkinsonisms. Ferroptosis is a form of non-apoptotic programmed cell death characterised by iron-induced lipid peroxidation and increased cellular reactive oxygen species (ROS) levels [11]. Although iron is indispensable due to its active involvement in several metabolic functions (e.g. enzymatic reactions, and oxygen transport), our cells are equipped with homeostatic mechanisms that help maintain iron concentrations and protect against ferroptosis [12]. Importantly, there are many metabolic pathways that may directly (or indirectly) regulate iron and redox homeostasis and thus affect the vulnerability of cells to ferroptosis. Several markers of the ferroptosis pathway (or associated metabolic mechanisms) have been identified to be controlling intracellular iron fluctuations [13] (Fig. 1). However, in ageing/age-related diseases, such as parkinsonism, these mechanisms become dysfunctional [14]; see also Table 1. Both PD and Tauopathy models have been linked to ferroptosis (Table 1), however, much less is known about ferroptotic cell death and its interaction with toxic protein inclusions, such as aS and tau. This is especially challenging when it comes to PSP/CBD as most research relating to tau is done in AD models, which contain other toxic proteinopathies and distinct tau isoforms than that of PSP/CBD, thus, there is a research gap in tau in the context of PSP/CBD.

**Table 1:** Ferroptosis-related marker expression in models of Parkinson's disease and Tauopathies.

Ferroptosis-related marker	Function	Expression/regulation	Model/technique
**Parkinson's disease**			
CoenzymeQ10	Antioxidant for protection against lipid peroxidation	↓	PD^b^ post-mortem brain [103], PD blood samples [106]
Ferritin	Iron-storing protein	↓ [78]/↑ [21]	PD post-mortem brain homogenates [78], proteomics in niagral tissues from PD post-mortem brains [21]
Ferroportin	Iron exporter (transmembrane) protein	↓	PD-like cell cultures [27,81], PD-induced rat model [27]
GPx4^b^	Rate limiting enzyme that prevents lipid peroxidation	↓	Post-mortem PD patient's SN^b^ [95], MPP+/MPTP^b^ -induced cell lines [96,97]
Iron	In PD, hypothesised to contribute to aS oligomerisation	↑	Human, rodent & cell culture models [101], iPSCs^b^ from PD patients & hESCs^b^ [60]
Lactotransferrin	Fe(III) importer into the intracellular space	↑	PD post-mortem brains (SN) [83]
NCOA4^b^	Breaks down ferritin to release intracellular iron	↑	PQ^b^ -induced mice and SHSY5Y cells [80]
NOX^b^	Potentiates ROS production	↑	MPTP-induced PD mouse model [36], dopaminergic neurons from PD patients and mouse models [36,37,39,40]
Nrf2^b^	Protects against oxidative stress (regulates transcription of antioxidants, such as GPx4)	↓^a^	Mouse model expressing human aS [109]
ROS^b^	Free radical production	↑	iPSCs from PD patients & rat primary cortical neurons [88]
*SLC7A11*^b^ (gene expression)	Encodes for one of the subunits in the Xc-b system	↓	Blood/saliva samples from PD patients [100], MPP+/MPTP-induced cell lines [96]
TfR1^b^	Intracellular iron uptake	↑	PD patient plasma [84]
4HNE^b^	Product of lipid peroxidation	↓	MPP+/MPTP-induced PC12 cells [54], post-mortem PD tissues [93,94]
**Tauopathy**			
ALOX^b^	Enter PUFA^b^ metabolism and potentiate lipid peroxidation	↑	Hippocampus of AD^b^ patients with NFTs^b^ [53], 3xTg-AD mouse model with tauopathies [52]
CoenzymeQ10	Antioxidant for protection against lipid peroxidation	↓^a^	P301S mice (tau + aggregation model) [131]
Ferritin	Iron-storing protein	↑	SWI^b^ images of CBD^b^ patients' SN [125]
Ferroportin	Iron exporter (transmembrane) protein	↓^a^	P301S mice (tau + aggregation model) [26]
Mitochondrial ferritin	Iron-storing protein in mitochondria	↑	SN of PSP^b^ patients [23]
Glutathione	Used to make GPx4 and prevent lipid peroxidation	↓	MSCs^b^ from PSP patients [28]
GPx4	Rate limiting enzyme that prevents lipid peroxidation	↓^a^	P301S mice (tau + aggregation model) [26]
Iron	In tauopathies, it is thought to lead to ↑ p-tau^b^	↑	AD patients' temporal cortex with NFTs [139]
NOX	Potentiates ROS production	↑	Human AD brains & mouse models with tauopathies [34,35], tau + cell cultures [38]
Nrf2	Protects against oxidative stress (regulates transcription of antioxidants, such as GPx4)	↑^a^	Various models with tau [133]
ROS/lipid peroxidation	Free radical production/oxidative damage leading to cell membrane damage	↑	MSCs from PSP patients [28]
TfR1	Intracellular iron uptake	↑^a^	P301S mice (tau + aggregation model) [26]
4HNE	Product of lipid peroxidation	↑	PSP neurons with abnormal tau accumulation [129], PSP patients' CSF [130]

Summary of the literature reviewed in text and the trends observed in the expression of ferroptosis-related markers in PD and Tauopathy models.

a Hypothesised, i.e. it means that the predicted direction of expression is extrapolated from observations in parkinsonism models treated with an anti-ferroptotic drug, compared to untreated; but no observation of control model compared to proteinopathy model.

b AD, Alzheimer's disease; CBD, Corticobasal degeneration; CoQ10, Coenzyme Q10; GPx4, Glutathione peroxidase 4; hESCs, Human embryonic stem cells; iPSCs, Induced pluripotent stem cells; LTF, Lactotransferrin; MPP+/MPTP, 1-Methyl-4-phenylpyridinium/1-methyl-4-phenyl-1,2,3,6-tetrahydropyridine; MSCs, Mesenchymal stem cells; SWI, susceptibility weighted imaging; NFTs, Neurofibrillary tangles; NOCA4, Nuclear receptor coactivator 4; NOX, Nicotinamide adenine dinucleotide phosphate oxidase; Nrf2, Nuclear factor erythroid 2-related factor 2; PD, Parkinson's disease; PQ, Paraquat; PSP, Progressive supranuclear palsy; ROS, Reactive oxygen species; SLC7A11, Solute carrier family 7 member 11 (Xc-subunit); SN, Substantia nigra; TFR1, Transferrin receptor 1; 4HNE, 4-hydroxynonenal.

Recently, valuable scientific contributions have successfully covered the ground of ferroptosis and its relation to neurodegeneration, amongst others, the review by Berndt et al. (2024) [15]. This review comprehensively revised the research that has been conducted in ferroptosis providing a novel overview of the new methodologies to measure ferroptotic cell death, presenting emerging chemical and pharmacological targets and also outlining how these pathways may be key in pathophysiology. This is further relevant when discussing emerging therapies and alternative targets that should be explored in future research, both of which are addressed in the present review. Although the link between ferroptosis and aS/tau pathology in PD and AD has been described, in the present review, we will emphasise the way in which the ferroptotic components connect to distinct parkinsonisms and their toxic inclusions. Approaching ferroptosis from a neuropathological angle, further helps to understand what novel treatment targets can be proposed and what experimental models can be used to conceptualise these. Thus, we will comprehensively cover these topics while also addressing the literature gap in parkinsonism and ferroptosis, and its relevance to understanding the disease.

**Current challenges in literature**:

• Is iron dyshomeostasis driving parkinsonism progression? And how does that differ from normal ageing?
• Are changes in the ferroptotic pathway a *cause* or *consequence* of pathology? Or independent of one another?
• What is the relationship between tau and ferroptosis in PSP/CBD? And how does it compare to aS in PD?

### 1.2 Iron (dys)homeostasis in ferroptosis

Ferroptosis is a form of non-apoptotic cell death, coined by Dixon and colleagues [11], characterised by iron accumulation-induced lipid peroxidation. Iron is normally found in red blood cells, bound to haemoglobin. Nevertheless, non-haemoglobin-bound iron is naturally absorbed in the gut, obtained via diet, but there are no proper excretion routes for excess iron [12]. As a result, our cells are equipped with homeostatic mechanisms that help maintain iron concentrations and protect against ferroptosis.

There are three main pathways via which iron dyshomeostasis/ferroptosis are thought to occur [13] (Fig. 1): 1) by free intracellular iron accumulation which contributes to the Fenton reaction, and formation of ROS and lipid peroxidation, as well as 2) when the Xc- system/glutathione peroxidase 4 (GPx4), which normally function to prevent ROS/lipid peroxidation [[16], [17], [18]], become dysfunctional, and finally, 3) the uncontrolled breakdown of intracellular lipids (lipophagy), further potentiating ROS/lipid peroxidation. All of these eventually lead to copious amounts of intracellular oxidative stress, triggering plasma membrane and organelle damage and, inevitably, ferroptotic cell death. These three main (dys)functional pathways can be broken down into more detailed mechanisms described below and depicted in Fig. 1:

#### 1.2.1 Free iron accumulation

As mentioned, non-haemoglobin-bound iron is obtained via diet as Fe(III) and reduced to Fe(II) to be absorbed in the gut [12]; alternatively, haemoglobin-bound iron can also be released from red blood cells as Fe(II) via heme group degradation by heme oxygenase 1 enzyme (HO-1). In neurons, Fe(III) can also enter cells by binding transferrin (TF) and consequently being up-taken via the transferrin receptor (TfR1) into the intracellular space [19] or, alternatively, Fe(III) may also be internalised by lactotransferrin (LTF) [20]. Either of these mechanisms leads to endosomal iron uptake, and the consequent STEAP3-induced reduction of Fe(III) to Fe(II), which then gets released to the cytoplasm via the divalent metal transporter 1 (DMT1, encoded by *SLC11A2*) [13].

In health and homeostasis, ferritin, an iron-storage protein, is thought to sequester this free intracellular iron and convert it back to Fe(III) preventing the toxic build-up of Fe(II) [21,22]. However, in less favourable conditions, ferritinophagy (i.e. the breakdown of ferritin), mediated by autophagy factors, such as ATG5/7 and LC3 [[22], [23], [24], [25]] may occur by the interaction with nuclear receptor co-activator 4 (NCOA4) leading to ferritin degradation and the release of reduced Fe(II) into the cytoplasm where it can accumulate [22,24,25]. Similar to ferritin, ferroportin-1 (FPN1, encoded by *SLC40A1*) also works to inhibit free iron [Fe(II)] build-up by exporting Fe(II) to the extracellular space; however, in neurodegeneration, FPN1 is thought to become dysfunctional therefore preventing the export of surplus iron [26,27]. Combined, the malfunctioning of these mechanisms that regulate intracellular free iron ultimately lead to an increase in Fe(II) and its consequent interaction with hydrogen peroxide via the Fenton reaction, eventually leading to increased ROS production and susceptibility to ferroptosis (Fig. 1, pink and yellow sections).

#### 1.2.2 Double edged sword - mitochondrial stress vs protection

One of the hallmarks of ferroptotic cell death is the observable reduction in mitochondrial size and the notorious decrease in the mitochondrial membrane surface area (i.e. lack of cristae) [11]. Mitochondrial autophagy (i.e. mitophagy) is one of the largest sources of ROS overproduction preceding ferroptosis [11,28,29]. Importantly, mitophagy involves the release of other proteins (e.g. cytochrome *c*) and the activation of caspases, neither of which is specifically observed during ferroptosis [11,30]. Interestingly, deletion of autophagy factors (e.g. ATG and LC3) mentioned above were seen to decrease ferroptosis susceptibility indicating that, while ferroptosis remains an overall non-apoptotic form of cell death, certain types of autophagy (e.g. ferritinophagy and mitophagy) may (indirectly) drive autophagy-dependent ferroptosis via their contribution to an iron and redox imbalance, ROS and lipid peroxidation.

In addition, in the mitochondrial membrane, Nicotinamide adenine dinucleotide phosphate (NADP) oxidases (NOX), as well as ferroptosis suppressor protein 1 (FSP1, encoded by the *AIFM2* gene) which oxidise NADP may trigger mitophagy [31]. It is noteworthy that FSP1 can also have a protective role in ferroptosis [32,33] which reinforces the idea that mitophagy may occur first as a protective mechanism to get rid of damage (for example, as an attempt to clear toxic protein inclusions); however, the uncontrolled mitochondrial breakdown may lead to toxic ROS release preceding ferroptosis. ROS overproduction, especially from NOX activation, is thought to become upregulated in parkinsonisms and its inhibition was seen to ameliorate the associated pathology [[34], [35], [36], [37], [38], [39], [40]]. This is because products of lipid peroxidation, such as 4-hydroxynonenal (4HNE) or malondialdehyde (MDA), can further activate NOX expression in the mitochondria, leading to a toxic positive feedback loop [41] (Fig. 1, yellow section). As a result, it has been hypothesised that NOX inhibitors may be beneficial in downplaying NOX-mediated ferroptosis in parkinsonisms [36,42]. In fact, a NOX1/4 specific inhibitor has been seen to prevent erastin-induced ferroptosis in cell cultures [11], suggesting the NOX family as a potential target for ferroptosis. Overall, it is clear that, although ferroptosis is uniquely distinguishable from normal autophagic cell death, selective autophagy within cells and NOX production seems to be key driver to ferroptosis susceptibility, and consequently, involved in promoting neurodegeneration progression.

Interestingly, exogenous mitochondrial transplants into HT-22 cells and primary cortical neurons of mice were shown to be neuroprotective and prevent RSL3-induced ferroptosis [43]. Moreover, these transplanted neurons did not show network fragmentation, nor damaging production of lipid peroxidation [43]. In line with this rationale, mitochondrial calcium is thought to also be a key modulator of mitochondrial function (i.e. modulating the shift from its neuroprotective function to becoming a ferroptosis contributor). In fact, ferroptosis is associated with overload of mitochondrial calcium accompanied by decreased mitochondrial respiration, which drive oxidative stress and ROS production. In a study by Marmolejo-Garza and colleagues [44], it was shown that inhibiting calcium uptake into the mitochondria of HT-22 and LUHMES cells protected them from ferroptosis, which is further indicative of the importance of mitochondria in the ferroptotic pathway. Thus, it is hypothesised that healthy mitochondria can have a neuroprotective effect and poses a promising therapeutic target. However, uncontrolled metabolic processes in mitochondria and a rise in its calcium levels may also facilitate ferroptosis in ageing and disease.

#### 1.2.3 Lipid peroxidation

Polyunsaturated fatty acids (PUFAs) are toxic via their incorporation into phospholipid membranes, with the aid of certain enzymes in the PUFA metabolic pathway, for example, acyl-CoA synthetase long-chain family member 4 (ACSL4) and lysophosphatidylcholine acyltransferase 3 (LPCAT3) [[45], [46], [47]]. Moreover, lipoxygenases (ALOXs) are iron-containing oxidative enzymes can also enter the PUFA metabolism and mediate lipid peroxidation by catalysing the last step in making toxic phospholipid-bound PUFAs [48]. Several *ALOX* genes have been identified in humans which may be driving ferroptosis. For example, *ALOX12* was seen to promote ferroptosis in cancer cells [49], while the *ALOX15* was seen to have a role in mediating RSL3-induced ferroptosis in various cells, including neurons [50], and *ALOX5* was also observed to be activated in erastin and RSL3-induced ferroptosis in cancer cells [51]. Thus, ALOX inhibitors pose a promising line of research to halt ferroptosis [18,52,53].

#### 1.2.4 Protective role of the Xc- system

Several pharmacological activators of ferroptosis pathways were recently discovered and act on various proteins associated with ferroptosis. For example, erastin acts via inhibition of the Xc- system and inhibition of voltage-dependent anion channels (VDAC2/3) in the mitochondria, while RSL3 works more downstream of the metabolic pathways leading to ferroptosis, by inhibiting the activity of GPx4 [11] (see Fig. 1). As such, the expression levels of the antiporter Xc- system is very important in preventing lipid peroxidation, and thus ferroptosis, during healthy states. The antiporter Xc- system is composed of two subunits (*SLC7A11* and *SLC3A2*) which exchange intracellular glutamate for extracellular cystine, which then gets fed into the glutathione (GSH) metabolism pathway (Fig. 1) [11]. That is, once cystine enters the cell, it gets first converted into cysteine and then GSH [11]. GPx4 plays an important role here as it can use GSH to detoxify peroxides and prevent lipid peroxidation [54]. Alternatively, another protective component acting in parallel to the Xc- system is non-mitochondrial Co-enzyme Q10 (CoQ10), which is a product of the mevalonate (MVA) pathway (illustrated in Fig. 1, pink/green sections) and can inhibit lipid peroxidation by FSP1 regulation [32,33]. FSP1, as mentioned, can inhibit ferroptosis by induction of (regulated) mitophagy. Therefore, the GSH and CoQ10 metabolism systems seem to be essential to preventing lipid peroxidation/ROS and maintain homeostasis, thereby decreasing the cell's sensitivity to ferroptosis.

#### 1.2.5 Protective antioxidant properties of Nrf2

Transcription induction of Nuclear factor erythroid 2-related factor 2 (Nrf2, encoded by the *NFE2L2* gene) not only can tightly regulate the activity of the xc-subunits [55], but studies have shown that is can also act on other key components downstream, such as glutathione synthase (GSS) and GPx4 [56,57], in order to confer protection against ferroptosis. Although Nrf2 properties still remain elusive, it seems to be a pivotal component of the pathway which may serve as a target to prevent ferroptosis in neurodegeneration, as discussed later in the review.

#### 1.2.6 SOS – repair mechanisms

Finally, as a last resort and defence mechanism when membranes have suffered lipid membrane disintegration, cells can activate repair pathways to dampen the damage and prevent ferroptotic cell death [58]. It has been shown that endosomal sorting complex required for transport (ESCRT)-III machinery activation can prevent ferroptosis by removing damaged components from the cell membrane [58]. Specifically, knockdown of ESCRT-III complex subunits was shown to increase erastin and RSL3-induced ferroptosis in cells [58], and FSP1 was also seen to activate ESCRT-III to prevent ferroptosis [59] (Fig. 1, green section). Moreover, calcium was seen to be pivotal in signalling this repair mechanism, which is also in line with previous research showing that calcium is a key regulator of ferroptosis via mitochondria [44,60,61]. Specifically, inhibition of mitochondrial calcium uptake or activation of small conductance calcium-activated potassium channels reduced mitochondrial calcium levels and prevented ferroptotic cell death [44,61,62]. Overall, ESCRT-III machinery, as well as cellular and mitochondrial calcium, seem to be important in rescuing cells with membrane damage; however, if damage is already too extensive when this machinery becomes activated, then it could further render cells more vulnerable to ferroptotic cell death [63].

In sum, several markers in the ferroptosis pathway have been identified to play a specific role in the cascade that controls intracellular iron fluctuations in healthy conditions (Fig. 1). However, in less favourable conditions, particularly seen in ageing and age-related diseases, these mechanisms become dysfunctional [14]. Thus, in the next sections we will discuss how ferroptosis is considered to be influencing age-related parkinsonisms.

## 2 Ferroptosis in parkinsonism

Ageing involves the progressive degeneration of several physiological processes, eventually leading to functional and cognitive decline, and inevitable death [64]. Chronic inflammation and the associated increase in ROS and oxidative stress have been proposed to be one of the main underlying causes of ageing and age-related disorders [64,65].

Furthermore, ageing is linked to the deterioration of cell systems that would otherwise sustain damage (e.g. protein quality control) [64], making individuals vulnerable to the excessive accumulation of pathological inclusions, such as misfolded aS in PD, and phosphorylated tau in PSP/CBD. Notably, some ferroptosis markers have been linked to tau and aS pathology in post-mortem human tissue, rodents and in cell lines (Table 1). Additionally, iron accumulation in the SN has been linked to PD progression [1] and excess iron was seen to co-localise with areas that accumulate tau [66], particularly in the basal ganglia (including SN *pars compacta*, SNc) [67]. Similarly, tauopathies in PSP/CBD were also found in the SNc [6,68]. All of the above processes point to the likely relationship between iron dyshomeostasis and the progression of pathology in parkinsonism, therefore shedding light on a new target for pharmacological intervention. However, before discussing these toxic inclusions in depth in their respective parkinsonisms, it is important to highlight the physiological functions of tau and aS in a healthy system. This is relevant later in the review when we discuss emerging therapies that aim to not only tackle the protein “toxic-gain-of-function” but also to preserve and potentiate the healthy functions of these proteins and truly progress in combating the deficits seen in parkinsonisms.

**Box 1: Physiological role of aS**.

The aS protein is encoded by the *SNCA* gene on chromosome 4 (human) [69]. While the complete physiological role of aS remains unclear, it appears that most of its known functions occur on cell membranes [70]. Specifically, its pre-synaptic involvement in neurotransmitter release and synaptic membrane curvature for exocytosis. Interestingly, recent evidence points to the role of certain types of aS oligomerisation (a-helical tetramer) to support nerve terminal normal function [70]. Primarily, physiological aS has been associated with monoamine (e.g. dopamine) metabolism and homeostasis at synapses, but also other general brain functions, such as, synaptic plasticity and learning [71]. It is challenging to study the aS toxic-gain-of-function damage in Parkinson's disease (PD) given that decreasing aS also creates deficits, alluding to its importance and vital role in health [72]. Interestingly, since aS also localises largely in the nucleus, several studies have argued for the role of aS in transcription, but it remains unclear if this function is physiologically relevant in humans [72,73]. It is however considered that aS binds DNA and histone proteins for transcriptional and epigenetic regulation [73]; and its level of aggregation also dictates the posttranslational modifications it suffers which may underlie pathological aS toxic-gain-of-function in disease [74].

**Box 2: Physiological role of tau**.

The tau protein is encoded by the *MAPT* gene on chromosome 17 (human). Various isoforms are found in the cortex of healthy adult brains, namely in neurons and glia. Neuronal tau is mainly located in axons where it interacts with microtubules, thereby stabilising them and connecting with cytoskeletal components (e.g. actin, neurofilaments). Tau knock-out (KO) studies also point towards a role in neuronal activity, neurogenesis, and synaptic plasticity. Overall, physiological tau is mainly involved in maintaining structural integrity within the CNS, transport along axons, and neuronal signalling [75]. Interestingly, it was recently observed that tau can aggregate and cause increased phosphorylated tau (p-tau) in neurons of hibernating animals, but this process is reversible upon arousal [76,77]. Investigators have proposed that increased p-tau in hibernation may be a neuroprotective response to reduced neuronal activity [77]. Thus, it has been hypothesised that p-tau may physiologically aggregate in “previously active” neurons in neurodegenerative brains, but with ageing, p-tau accumulates into toxic tangles via several posttranslational modifications [76,77].

## 3 Ferroptosis and aS in Parkinson's disease

**Fig. 2:**
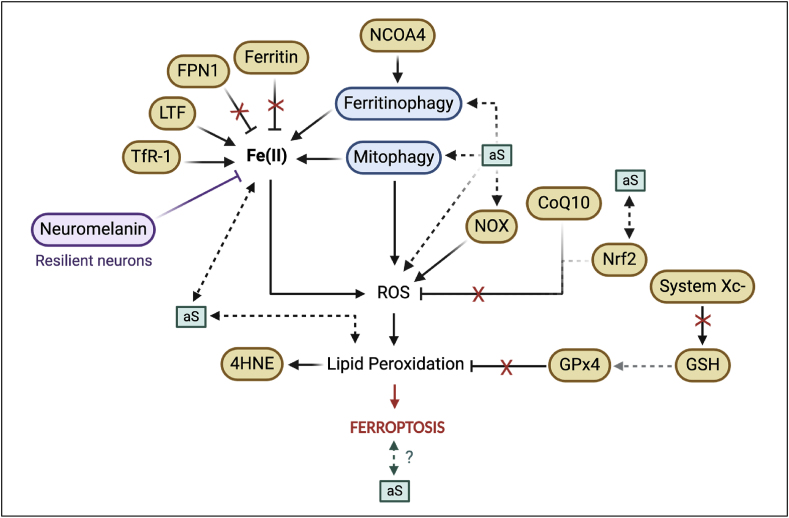
Ferroptosis-related events in the presence of aS (α-synuclein) pathology. Several markers of ferroptosis have been identified to be possibly contributing towards ferroptotic cell death with aS due to 1) increasing intracellular iron (LTF, TfR1, NCOA4, and malfunctioning of ferritin and FPN1), 2) increasing ROS (NOX, and disinhibition of CoQ10/Nrf2) and 3) enhancing lipid peroxidation (blockage of the Xc- system and GPx4 activity, and consequently, increased 4HNE production); some of these markers have been directly linked to pathology (aS). Neuromelanin, although not a marker of ferroptosis, is thought to work as an iron chelator and thus, protect against ferroptosis, as it is observed in resilient neurons (i.e. surviving neurons from diseased patients). Dotted lines represent indirect observations and hypothesised relationships from the literature reviewed in text. Xc-, Glutamate/cystine antiporter system.

As previously mentioned, intracellular aS in the SNc is associated with the pathology of PD [6]. Multiple experimental models have been used to study and describe ferroptosis in PD progression (Table 1) (Fig. 2). However, while many ferroptosis-related markers have been observed to be changed in models of PD, not all of these markers have been studied in the context of aS specifically. Fig. 2 illustrates what we know about the role of aS aggregates in ferroptosis, which further provides an overview of the research gaps from the toxic proteinopathy angle. Thus, in this part of the review, we will discuss, in the same order as previously, how some of the above mentioned ferroptosis-related processes may be related to aS pathology.

### 3.1 Free iron accumulation

Ferritin was observed to be decreased in post-mortem PD brain tissue homogenates [78] (Fig. 2), whereas another patient study reported an increase in ferritin in post-mortem nigral tissues from PD patients [21]. However, it is important to consider that, by the time of a PD diagnosis, 50–90 % of dopaminergic neurones are already lost [79]. These reports comparing ferroptosis marker expression in PD patients to age-matched controls may be conflicting and skewed if neuronal density is not considered. Additionally, NCOA4 was upregulated in a PD cell culture model [80], likely triggering degradation of ferritin and contributing towards elevated intracellular iron levels. Similarly, FPN1 was observed to be downregulated in PD cell culture models [27,81] and in a PD-induced rat model [27] (Fig. 2). However, another study argued that FPN1 becomes rather internalised in disease and rendered dysfunctional due to its absence from the membrane, but its expression is increased intracellularly as a result [82]. It is tempting to speculate that increases observed in NCOA4 may be related to decreased levels of ferritin due to its degradation which, combined with dysfunctional FPN1, lead to elevated free iron intracellularly, rendering cells vulnerable to ferroptotic cell death.

Free iron is also thought to accumulate in PD tissues via an increased influx in disease [83]. In fact, levels of LTF, a protein that internalises Fe(III), were seen to be upregulated in resilient neurons of PD patients which correlated with an observed increase in Fe(III) in the SN of PD cases, compared to controls [83]. This is indicative of more Fe(III) entering the cells under PD(-like) conditions and alluding to the likely active role of LTF in pathological changes and neurodegeneration, via a pathway upstream of ferroptotic cell death. Moreover, another importer of iron, TfR1, was seen to be increased in the plasma of PD patients with high degree of tremors [84], suggesting a link between intracellular iron load and exacerbation of motor symptomology. These findings therefore highlight the importance of studying how targeting iron may interfere with mechanisms of aS pathology and thus, serve as a therapy in PD. While these studies were done in the SN of PD tissues, not all cells contain aS at the time of death, thus, although there is an indication that aS may be affecting these changes given the tissue used, these investigations do not address the role/interaction of the toxic inclusion itself which can be achieved by, for example, quantification of the co-localisation of these markers with aS. Interestingly, as aS is a protein that highly interacts with membranes [85], this suggests a possible link between the ferroptotic components that are also membrane-bound, such as mitochondrial membrane proteins (NOXs) reviewed in the next paragraph of the review, but also FPN1 here mentioned. Later in the review, we indeed dive deeper into the interplay of iron accumulation (in part mediated by FPN1) and its possible role in the aggregation of aS in the progression of PD.

### 3.2 Double edged sword - mitochondrial stress vs protection

NOX is involved in potentiating ROS production in mitochondria and, as dopaminergic neurons are especially vulnerable to oxidative stress [86], it is considered that NOX could be more active in these cells [37,39,40]. In fact, PD genetic factors (e.g. *PINK1*) have been linked to NOX regulation [87], namely, an increase in NOX was observed in the MPTP-induced PD mouse model, whereas MTPT-induced NOX-knockout (KO) mice showed decreased oxidative stress and neuronal loss [36]. Moreover, specific NOX isoforms, such as NOX-1, -2 and -4, were upregulated in dopaminergic neurons of PD patients and/or mouse models [37,39,40], and this NOX activity was, in part, linked to aS [39,88]. Interestingly, treatment with a specific NOX-1, -2 and -4 inhibitor rescued the effects mediated by aS-fibrils injected mice and N27 rat dopaminergic cells, paralleled to a decrease in aS oligomerisation and oxidative stress [42]. This was also seen in human mesencephalic progenitor cells, whereby NOX1 and aS expression were increased in a paraquat (PQ)-induced PD assay, as well as increased aS aggregates observed in a PD model of PQ-induced rats with a NOX1 KO in the SN [89]. Thus, NOX inhibition may rescue aS-containing cells in PD.

Concomitantly, dopaminergic cell death in PD patients has been associated with an increase in calcium and consequent mitochondrial oxidative stress and mitophagy. In line with this, calcium oscillations are known to be important in dopaminergic neuron pacemaking, and disruptions could underlie susceptibility to neurodegeneration [90]. Interestingly, while aS accumulation was seen to increase calcium transients in mitochondria of HeLa cells [91], it was also observed that *PINK1*, one of the best characterised PD mutations, plays a role in mediating mitochondrial calcium efflux [92]. Thus, *PINK1* deficiency, characteristic of PD, may induce calcium overload in mitochondria triggering its dysfunction and increased ROS [90]. Therefore, and as mentioned earlier, healthy mitochondria are important for neuroprotection [43], but its dysfunction can drive toxic cellular pathways and possibly contribute to ferroptosis.

### 3.3 Lipid peroxidation

Another well-recognised ferroptosis marker is 4HNE, a direct by-product of lipid peroxidation, which has been shown to be upregulated in post-mortem PD tissues [93,94], and in MPP + -induced cell death in various cell lines [54]. Since lipid peroxidation is thought to be a critical cellular event preceding ferroptotic cell death, these findings suggest that cells from PD patients or cell lines chemically induced to mimic PD characteristics, are more susceptible to ferroptosis.

### 3.4 Protective role of the Xc- system

The enzyme that inhibits lipid peroxidation, GPx4 (Fig. 1, green section), was seen to be decreased in post-mortem tissues of PD patients’ SN [95], as well as in MPP^+^/MPTP-induced cell lines [96,97]. It is noteworthy that Bellinger and colleagues [95] reported that, while GPx4 was decreased in PD tissues, GPx4 could interact with aS, and associate with neuromelanin. Interestingly, GPx4 levels were found, in fact, increased relative to overall cell density, as a function of surviving SN neurones. Thus, as GPx4 was also seen to co-localise with neuromelanin [95], neuronal expression of neuromelanin and GPx4 could be acting as neuroprotective when there is increased neurodegeneration [95,98,99]. This means that, during neurodegeneration and loss of neurones (and thus, neuromelanin), GPx4 may also be reduced as a result. Concomitantly, the *SLC7A11* gene, which encodes for the Xc- system, regulator of GPx4, was seen to be less expressed in PD tissues [100], which is in line with the overall GPx4 decrease observed when neuronal density is not taken into account [95]. These findings combined further solidify the possible neuroprotective role of the xc-/GPx4 system in (preventing) PD (Fig. 2), by decreasing oxidative stress and neuronal cell death [100].

**Box 3: Does neuromelanin confer resilience to cell death?**

Neuromelanin is a dark pigment, amongst others, found in the substantia nigra *pars compacta* (SNc) dopaminergic neurons and its loss is a very prominent feature of Parkinson's disease (PD) and other forms of parkinsonism. Neuromelanin has also been reported to work as a metal chelator, namely of iron, serving as a neuroprotective agent by suppressing iron redox cycling in vesicles [101]. Thus, it is tempting to speculate that surviving aS-containing neurons in PD are resilient, and not yet dead, due to the protective presence of neuromelanin, and its association with GPx4, an inhibitor of lipid peroxidation [95].

As previously mentioned, CoQ10 could mediate protection against lipid peroxidation (Fig. 2). Indeed, studies have shown that CoQ10 can be neuroprotective against PD-related deficits in MTPT-treated mice [102]. Similarly, PD patients seem to have decreased CoQ10 in post-mortem brains [103], platelets [104,105] and in blood samples [106]. It is noteworthy, however, that this CoQ10 deficiency was not seen in the SN [103]. Concomitantly, recent studies have questioned the ability of CoQ10 supplementation in improving PD (dopaminergic) neuronal deficits, as a result of its limiting benefits [107,108]. In conclusion, although targeting CoQ10 has shown early promise in the treatment of PD, it is unclear if CoQ10 impact is negligible in dopaminergic neurons, which are largely affected in PD. On the other hand, the Xc- system and its downstream activity, highly characterised in ferroptosis, seem to regulate neuronal cell death in PD, which could indicate that neuronal cell death observed in PD is a result of ferroptosis; thus, targeting ferroptosis-related markers pose a viable route of investigation for slowing down/stopping PD progression.

### 3.5 Protective antioxidant properties of Nrf2

Several lines of evidence suggest that Nrf2, a transcription factor that regulates NRF2 protein antioxidant activity, has a protective role against oxidative stress, and this process might be impaired in PD. In a Nrf2-KO mouse model expressing human aS in the midbrain, KO mice had increased degeneration of nigral dopaminergic neurons, exacerbated aS aggregation and more Lewy bodies (LB), as well as more pronounced neuroinflammation [109]. Furthermore, pharmacological activation of Nrf2 in the SN had a protective role on dopaminergic neurons of Nrf2-KO mice with human aS. Similarly, in post-mortem PD patients it was observed that proteins associated with NRF2 expression were trapped within LB formations, rendering Nrf2 function presumably negligible in these tissues [109]. Interestingly, Nrf2 is more expressed in the nucleus of nigral dopaminergic neurons in PD, which is argued to be a result of a higher demand for increased transcription of antioxidant enzymes in neurodegeneration [110].

It is also important to note that post-mortem tissues do not adequately translate the stage of the Nrf2 function in disease and thus, unclear to conclude if results observed are in fact characteristic of the disease progression or a temporary compensatory mechanism [111]. For example, in induced pluripotent stem cell (iPSC)-derived neurons from PD patients, increased oxidative stress was paralleled to an increased activity of the Nrf2 pathway [112]. However, other models have shown that iron promotes aS aggregation via Nrf2 inhibition [113] and genetic deletion of Nrf2 enhanced aS toxicity [109]. Together, these studies suggest a relationship of aS aggregation and Nrf2, however, Nrf2 profiles seem to change throughout disease course and targeting this pathway is likely highly dependent of a specific time window of treatment.

Here we presented the literature focused on the fluctuations in specific ferroptosis-related markers in the context of PD. In the following segment of the review, we explore in more detail the current evidence that tries to draw a link between iron-related events and aS aggregation. We also discuss how these findings can be helpful in developing novel therapeutic avenues in the treatment of PD.

### 3.6 The interplay of aS and iron metabolism

Iron accumulation and ferroptosis-related events could contribute to aS oligomerisation and its consequent accumulation [101]. However, the presence of aS has also been hypothesised to be partially responsible for iron dyshomeostasis via mitophagy and ferritinophagy [101]. Furthermore, aS oligomers were seen to become more internalised in oxidised membranes, compared to non-oxidised membranes, suggesting that aS internalisation occurs after lipid peroxidation has taken place [60]. It was further observed by Angelova and colleagues [60] that administration of a ferroptosis inhibitor (Ferrostatin-1, Fer-1, Fig. 1) and an iron chelator (Desferoxamine, DFO) reduced aS oligomer-induced cell death. Therefore, it is tempting to speculate that there may be a bi-modal relationship between aS accumulation leading to subsequent iron build-up and ferroptosis but, in parallel, lipid peroxidation due to iron accumulation further increasing the ease with which aS internalises into cells that are still alive (Fig. 2).

As reviewed in this PD section, although much research has been conducted in the field of PD and ferroptosis using several models, not all of them cover in detail what could be happening at the toxic aS level and how proteinopathy may be affecting ferroptosis pathways. As discussed, iron could be a key driver of aS oligomerisation into toxic aggregates, and it is possible that FPN1, an exporter of iron, may be related to this phenomenon by impairing iron export. Similarly, it was seen that mitophagy may influence this metabolic process, and this is in line with the revisions made regarding the function of the NOXs at the level of the mitochondrial membrane, introducing several layers of the metabolic nodes that are upstream of ferroptosis and may serve as potential targets. Interestingly, as dopaminergic neurons are highly energetically demanding and specifically vulnerable to oxidative damage, it is increasingly evident that ferroptosis may be having an effect in progression of PD – but not enough evidence can explain if this is dependent or happening in parallel to aggregation of misfolded aS.

### 3.7 Emerging therapies in PD

The misfolding and toxic aggregation of aS represent the major hallmarks in PD at a cellular level. As such, it is not surprising that a lot of emerging research has circled around the elimination of these toxic inclusions. Recent clinical trials have thus focused on the removal of aS by using monoclonal antibodies in PD patients (PRX002 phase 1 and ongoing phase 2 - NCT03100149) [114] and others by preventing aS aggregation in healthy subjects (NPT200-11 phase 1 - NCT02606682). Despite these innovative approaches, vaccine preliminary results have been so far promising in determining the toxicity/tolerability of the compounds but with ambiguous results regrading symptom improvement in treated individuals. Of note, the PD trials were targeted at early-stage PD patients (according to the Hoehn and Yahr scale), however, early-stage in the clinic means some manifestations of motor symptoms, at which point, from a neuropathological point of view, aS has spread through several parts of the brain and has reached the dopaminergic centre, having now likely internalised into dopaminergic neurons and caused the death of others. This reiterates that despite knowing that dopaminergic neurons are specifically vulnerable in parkinsonism and that proteinopathies are thought to be the culprit for part of its progression, it is clear that generally targeting these could be highly limited to a narrow time window since symptoms appear when pathology spread is already detrimental and neuronal loss/impairment has begun.

From this review it is clear that although further research is encouraged to understand the interplay of PD pathology and iron-related events, ferroptosis seems to play an important role in PD progression, perhaps from early stages of disease development. In fact, a clinical trial showed promising results in the use of DFO in the treatment of PD and elucidated the potential of targeting the ferroptotic pathway to treat and/or prevent PD [115]. Interestingly, two clinical trials from the same research group have challenged the therapeutic efficacy of iron chelators [116,117]. Earlier in 2014, in a pilot study, they observed that early-on oral administration of deferiprone (DFP, a labile iron chelator) in PD patients delayed disease progression and decreased iron deposits in the SN [117]. However, in a later study, Devos and colleagues [116], in a phase 2 trial, reported that PD patients receiving DFP actually had worsened symptoms and adverse effects. It is noteworthy that in the 2014 trial, all patients were on a stabilised treatment course of dopamine agonists and/or l-dopa, while in 2022, the cohort of newly diagnosed patients had never received any treatment for the disease.

The success in the pilot study [117] may be indicative of a co-treatment effect of DFP with dopamine agonists drugs. This is not surprising given the complex nature of the disease. For example, given that dopamine agonists are established to be crucial in maintaining dopaminergic neuron integrity, it is likely that this needs to be assured in order to observe a successful effect of iron chelation as a way to prolong the treatment effects of these treatments. In a similar context, it is possible that iron chelation is more effective in earlier stages of disease indeed due to the fact that there is less dopaminergic neuronal loss. Thus, despite targeting several markers of ferroptosis or targeting free iron directly seeming promising, it is also important to note that many pathways have been proposed to be involved in ferroptosis-related events (depicted in Fig. 1) and/or PD. Therefore, it is not surprising that inhibiting only one component may not produce outstanding results, which, in turn, may be demoralising the use of anti-ferroptotic drugs to treat PD/neurodegeneration. Consequently, it is critical for future research to address multiple targets at once and evaluate the effects of a combination of drugs to possibly optimise efficacy and further explore ways of targeting ferroptosis as a therapy to PD.

## 4 Ferroptosis and tau in PSP/CBD

Atypical parkinsonian syndromes, such as PSP and CBD, account for 5–7% of parkinsonisms, but are often undistinguishable from PD in the clinic [4]. Although the co-localisation of some, but not all, ferroptosis-related markers with tau pathology has been studied in Alzheimer's Disease (AD) [118], the expression of these markers in the presence of tau in the context of parkinsonism is scarce. It is noteworthy that AD tissue also bears Amyloid-beta (Aβ) pathology, therefore, making it more challenging to propose how ferroptosis-related markers would behave in the unique presence of tauopathies in PSP/CBD.

Studies have shown that iron deposition correlates with pathology accumulation [[119], [120], [121]]. It was seen that oxidation of Fe(II) to Fe(III) redox cycling seem to be particularly toxic in the brain and potentiate ferroptosis [121,122], as well as what could be driving the aggregation of soluble tau (s-tau) into toxic neurofibrillary tangles (NFTs) [121]. In fact, converting Fe(III) to Fe(II) reversed the aggregation of hyperphosphorylated tau in NFTs back into s-tau [121].

**Fig. 3:**
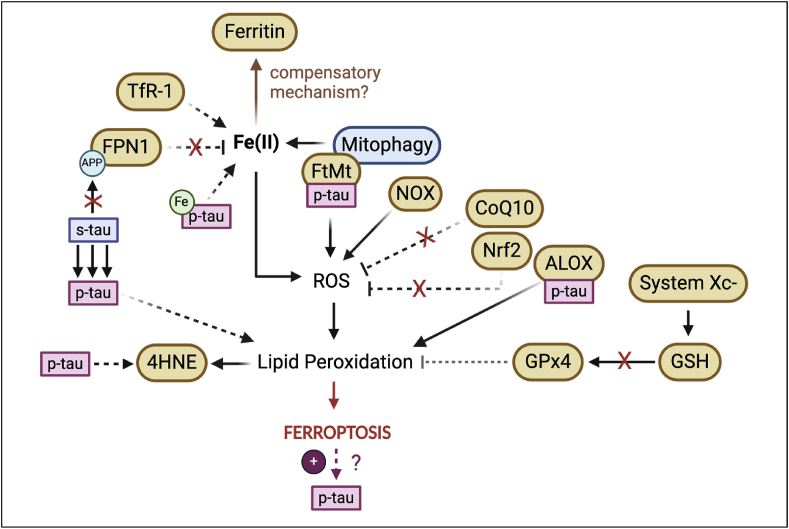
Ferroptosis-related events in the presence of tauopathies. Several markers of ferroptosis have been identified to be possibly contributing towards ferroptotic cell death with tau due to 1) increasing intracellular iron (TfR1, Fe release from FtMt due to mitophagy, and malfunctioning of FPN1 due to lack of soluble tau), 2) elevated ROS (NOX, disinhibition of CoQ10 and Nrf2 activity) and 3) enhanced lipid peroxidation (increased ALOX activity, decreased GSH, and consequently, increased 4HNE production); some of these markers have been directly linked to pathology (phosphorylated tau, p-tau). Ferritin has been reported to be increased which could be acting as a primary compensatory mechanism to store surplus iron. Dotted lines represent indirect observations and hypothesised relationships from literature reviewed in text. Xc-, Glutamate/cystine antiporter system.

Furthermore, Fe(III) has been seen to accumulate in NFTs which, combined with increased ROS, leads to ferroptosis and further hyperphosphorylation of tau [121] (Fig. 3). Concomitantly, emerging imaging techniques have revealed that PSP patients show increased iron deposition in subcortical nuclei, including the SN [120].

**Box 4: What distinguishes PSP from CBD?**

**Fig. 4:**
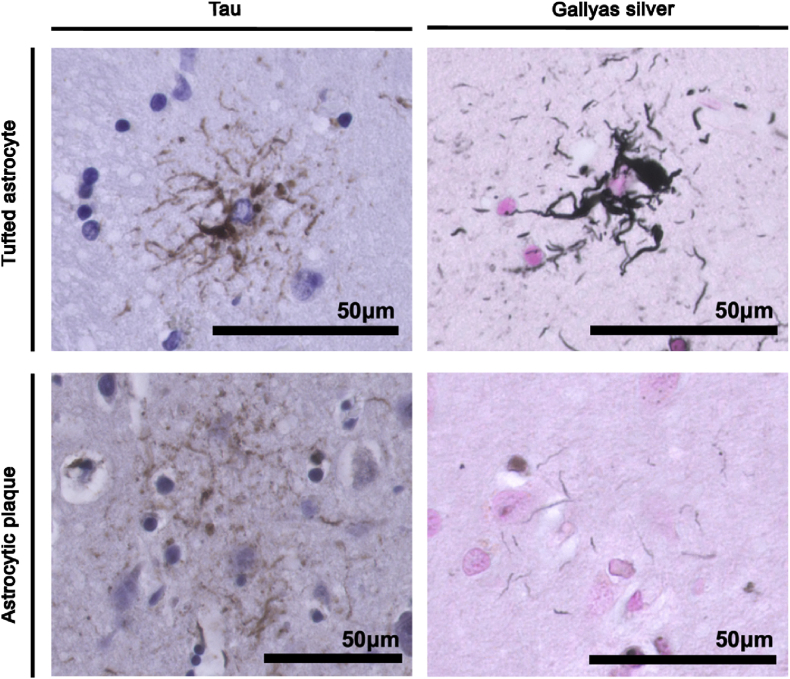
Histological images representative of differences in astrocytic morphologies in PSP and CBD. On the top *left*, an example of a tufted astrocytes typical from PSP patients, stained against tau (anti-AT8) and, on the *right*, the corresponding Gallyas silver stain (to label glial tau inclusion pathologies). On the bottom *left*, an example of an astrocytic plaque typical from CBD patients, stained against tau (anti-AT8) and, on the *right*, the Gallyas silver stain. All images provided have been obtained at the histology laboratory of the University of Groningen Medical Centre (UMCG).

Progressive supranuclear palsy (PSP) and Corticobasal degeneration (CBD) are both associated with filamentous tau inclusions, predominantly of the four-repeat tau protein, spanning both neurons and glia [123]. The main difference that distinguishes both diseases pathologically are astrocytic lesions [123]. PSP is characterised by tufted astrocytes, which take on a radial-like structure, with thin and long branches, and fibrous appearance [124] (Fig. 4). Conversely, CBD exhibits astrocytic plaques which take on a corona-like shape, with fuzzy and short processes [124] (Fig. 4). Additionally, in PSP, tau-affected neurons have dense and compact filaments (NFTs), while in CBD, tau is more commonly seen as a wispy and fine inclusion within the some [123]. Although there is a large pathological and clinical overlap between PSP and CBD, there are minor differences that can be used to continue to treat them as separate disorders. Nonetheless, in the study of tau-related parkinsonisms, their similarities allow us to pool them to study the relationship between tau and ferroptosis.

Moreover, an early CBD case study found that neurons in the SNc were pale and lacking neuromelanin, accompanied by extensive atrophy, neuronal loss, elevated NFTs and striking iron accumulation throughout the CNS [119], seen across five brains of CBD patients. Another CBD case study with neuroimaging observed that CBD brains have more bound-iron deposition, especially within the SN, and this can be detectable in patients using susceptible weighted imaging (SWI) which enables iron-containing compounds, such as ferritin, to be used in contrast during magnetic resonance imaging (MRI) [125]. However, as this technique measures bound iron and not free iron, conclusions about ferroptosis susceptibility are therefore limited. It is noteworthy that the CBD patients studied by Mizuno and colleagues [119] also showed extensive similarities to PSP neuropathology which emphasises the resemblances between PSP and CBD, hence why it is important to study these two disorders together and uncover treatment targets that could benefit patients suffering from either.

Thus, in order to investigate how PSP/CBD patients could benefit from possible iron-targeting approaches, we will delve into the research regarding ferroptosis-related markers and tau in the same orderly manner as read in previous segments of the present review.

### 4.1 Free iron accumulation

FPN1 was shown to be decreased in the tissues of P301S mice (a mouse model with tau aggregation pathology) (Fig. 3) compared to the treated group administered with α-Lipoic acid (LA, Fig. 1), a drug with antioxidant and iron chelating properties [26]. It was further observed that mice treated with the anti-ferroptotic drug had decreased TfR1 protein (Fig. 3) paralleled to decreased p-tau, as well as improved learning and memory [26]. Thus, treating mice with α-Lipoic acid not only changed ferroptosis marker expression but also prevented tau oligomerisation and cognitive decline.

Another marker that has been explored in PSP/CBD is ferritin. Mitochondrial ferritin (FtMt, Fig. 3), an iron store within mitochondria, was shown to be increased in the SN of PSP patients and this was related to cell damage [23]. This was further associated with an increase in p-tau which is thought to induce mitophagy and contribute to ferroptotic cell death [23]; p-tau was also seen to co-localise with FtMt previously [23]. Interestingly, Abu Bakar and colleagues [23] also detected that an autophagy marker, LC3, co-localised with FtMt in conditions of increased oxidative stress [23,126]. Thus, FtMt might have a protective function by sequestering iron but, under stress conditions, mitophagy could override this function [23]. Concomitantly, in a recently published CBD case study [125], SWI showed that iron-containing proteins, such as ferritin, were deposited in deep brain nuclei, including the SN, which could be indicative of a compensatory mechanism for the tau-associated increase in iron content (Fig. 3). Overall, it is apparent that when iron builds up intracellularly, ferritin (and FtMt) works as an initial repair mechanism to sequester this surplus iron. However, as oxidative stress increases, this may cause selective autophagy (e.g. mitophagy) and contribute towards further ROS production.

### 4.2 Double edged sword - mitochondrial stress vs protection

Tau aggregation was further seen to activate NOX pathway machinery [38], especially NOX-4 expression which is widely expressed in neurons, and reported to be increased in human AD brains with hyperphosphorylated tau and mouse models with tauopathies [35]. In fact, NOX-4 and -2 downregulation/deficiency were seen to decrease tauopathy load in mice [34,35]. Therefore, oxidative stress seems to be driven by tau aggregation which, in turn, may be contributing towards the consequent malfunctioning of other ferroptosis-related pathways, such as iron release from FtMt. Furthermore, it was observed in vitro that aggregated tau fibrils can modify calcium transient currents in primary neuronal cultures [38]. In fact, this increased cytosolic calcium further activated NOX pathways, leading to more ROS production [127]. These calcium and NOX events were seen to be recapitulated by the incorporation of exogenous tau fibrils into membranes and ultimately resulting in neuronal cell death/dysfunction [38]. Moreover, the increased cytosolic calcium oscillations were also accompanied by enhanced mitochondrial uptake of calcium, and consequently faster mitochondrial depolarisation and cell death vulnerability. Concomitantly, in iPSC neurons from patients with frontotemporal lobe dementia, with a *MAPT* tau mutation, were also seen to be more prone to mitochondrial ROS production, but this was prevented with antioxidants [128].

These findings support the idea that ferroptosis may also be targeted using mitochondrial antioxidants and agents that prevent excessive uptake of mitochondrial calcium, thereby reducing ferroptosis vulnerability via moderation of mitochondrial bioenergetics.

### 4.3 Lipid peroxidation

In a different study focused on PSP, neurons with abnormal tau accumulation were seen to be more immunoreactive to 4HNE and the marker's expression co-localised with tau inclusions [129]. Interestingly, although tau-positive neurons from PSP patients had increased 4HNE expression compared to PSP tau-negative neurons and neurons from control patients, PSP tau-negative neurons still expressed high 4HNE levels compared to controls, which is indicative of an overall effect of disease state on ferroptosis, beyond just pathology [129]. This, combined with an increase in oxidative markers, suggests that ferroptosis may even precede tau aggregation or be parallel/independent to it [129]. Furthermore, increased 4HNE (Fig. 3) was observed in cerebrospinal fluid (CSF) from PSP patients [130]. Interestingly, administration of a drug that prevents lipid peroxidation (RT001, Fig. 1) rescued ROS and lipid peroxidation in mesenchymal stem cells (MSCs) from PSP patients [28]. Overall, these independent investigations have in common the fact that using lipid peroxidation as a target, can rescue oxidative stress and rescue nerve cell death in Tauopathy models.

Similarly, ALOX enzymes, part of the toxic PUFA metabolism, were seen to be increased in the hippocampus of AD subjects with NFT pathology, while largely absent from healthy controls [53]. Ikonomovic and colleagues [53] further observed that this ALOX upregulation also co-localised with NFTs. Concomitantly, ALOX inhibition was shown to reduce tau-related proteins and overall p-tau load in the triple transgenic (3xTg) mouse model of AD with tauopathies [52]. Thus, this reinforces the relationship between tau aggregation and ferroptosis susceptibility which may be driving PSP/CBD progression.

### 4.4 Protective role of the Xc- system

GPx4 was seen to be increased significantly in animals treated with the iron chelator, LA [26]. Decreased levels of GSH, an important antioxidant (Fig. 1, green section), were seen in PSP MSCs but this was also rescued by an anti-ferroptotic/anti-lipid peroxidation drug, RT001 [28] (Fig. 1, yellow section). Additionally, CoQ10 activity, a secondary line of protection against ferroptosis, has been hypothesised to be lower in tauopathies [131] (Fig. 3). Concomitantly, it was observed that increasing CoQ10 improved neurodegenerative-like phenotypes, as well as reduced oxidative stress and lipid peroxidation, in the tauopathy P301S mouse model [131]. However, similar to PD studies, targeting CoQ10 alone may be limiting in the treatment of neurodegeneration. In line with this, a recent study observed that, although patients with dementia tend to have an overall decreased level of CoQ10, this was not associated with tau aggregation [132], further solidifying that multiple systems may be failing during disease.

### 4.5 Protective antioxidant properties of Nrf2

Nrf2 activity decreases during ageing and is thought to be driving neurodegeneration through impaired detoxification [133]. It has been shown that Nrf2 pathway activation can aid in the reduction of tau phosphorylation through the activation of autophagy [134] which has been shown to clear tau in ageing [135]. Furthermore, in a P301S mouse model overexpressing tau, Nrf2 loss aggravated hind-limb paralysis, memory and neuronal loss [136]. Conversely, post-mortem brains from PSP patients showed increased Nrf2 levels, which could be indicative of a compensatory mechanism to combat cell death in surviving neurons [137]. Similarly, HO-1 (an antioxidant enzyme activated by Nrf2) was also seen to be increased in CBD patient tissues [138]. These results suggest that oxidative processes are likely occurring in PSP/CBD tissues, paralleled to tau pathology. However, Nrf2 pathway activation observed in surviving neurons could provide a beneficial effect to compensate for the excessive oxidative stress and to prevent cell death.

Combined, the markers reviewed in this section indicate that there are many possible ways to successfully target the ferroptosis pathway in tau-related diseases, and importantly, that ferroptosis-related markers are fundamentally changed in tauopathy-mediated neurodegeneration. However, it also elucidates the complexity of this type of cell death and how we should seek in-depth research into the molecular aspects of the interplay between cellular mechanisms, instead of focusing on a single marker of ferroptosis. Furthermore, it is pivotal to understand how tauopathy formation/aggravation relates to iron imbalance observed in patients, animals and cell models reviewed. In the following section, several lines of research are presented to argue the currently enigmatic relationship of iron-associated tau pathophysiology and tau-related parkinsonism.

### 4.6 Controversy of tauopathies and iron-related events

The literature reviewed this far presents evidence in support of ferroptosis pathway in PSP/CBD tissues, and the association of these iron events with tau; however, other studies have shown conflicting data regarding tauopathies. It has been argued that iron accumulates in the temporal cortex of AD patients, but these deposits only correlated moderately with NFTs, whereas symptomology/clinical diagnosis was strongly correlated with iron in the temporal lobe of the same patients [139]. Therefore, Ayton et al. [122,139] hypothesised that iron may potentiate degeneration when pathology is already present and severe, as parallel events happening independently, despite research having argued for a causal relationship between tau and iron-related events [122]. Additionally, in a large cohort study, an increase in iron biomarkers was associated with clinical progression of AD but not pathology, thus hypothesising that iron is required in the *probability* of degeneration in AD but not the main driver [122]. Furthermore, redox cycling of Fe(II) to Fe(III) was seen as the toxic and dangerous event driving ferroptotic cell death, rather than overall increase in iron, further emphasising the role of iron as a mediator of AD susceptibility [122]. Therefore, these data indicate that biochemical changes secondary to proteinopathies could further contribute to iron burden and worsen the disease state [122].

It was further observed that tau-KO mice, which show age-dependent parkinsonism phenotypes, had increased iron build-up coupled to increased neuronal cell loss in the SN. It was proposed by Lei and colleagues [140] that amyloid precursor protein (APP) ferroxidase, an enzyme needed to move FPN1 to the cell membrane, is dysfunctional in neurodegeneration, therefore leading to the intracellular accumulation of iron as it does not get exported (Fig. 3). In primary neuronal cultures of the tau-KO mice it was further observed that this loss in soluble tau impaired FPN1 trafficking [140]. The absence of tau prevented its interaction with APP and consequently, APP movement to the cell surface where it would normally co-localise with FPN1 [140]. Thus, s-tau might be lost to form NFTs in disease and the subsequent lack of s-tau due to its oligomerisation could explain the low iron efflux (Fig. 3) observed in tau-KO neurons [140], deeming s-tau functional for disease prevention. In line with this rationale, it is thus believed that only p-tau, and not s-tau, is toxic in neurodegeneration and iron events [140]. Emerging research into treatments addressed in the following section also focus on this aspect.

Remarkably, ferroptosis and tau have been studied mostly in the context of AD. However, AD shows differential tau isoform/filamentous pathology to that of PSP/CBD patients, and even other Tauopathies, such as Pick's disease are all characterised by distinct isoforms [141]. This is challenging not only when dealing with the translation of tau research in AD to PSP/CBD, but also when generating research models of tauopathy and tau-related parkinsonism. It is also important to consider that these animal models are mainly chemically or transgenic models, with several modifications that do not replicate the tau isoforms that specifically affect PSP/CBD, posing a critical obstacle in the pursuit of fundamental research in interplay of tau and ferroptosis in Tauopathy. In order to further the research in this field from a neuropathological angle, the use of inoculation models could be explored for PSP/CBD and PD animal model. Inoculation is a technique in which human brain homogenates from patients are injected into the brains of transgenic mice, allowing the reproduction of human-like pathology in these animals, which very accurately mimics the permissive-templating transcellular spread of proteinopathy seen in humans, and recapitulates characteristics of disease not seen with standard transgenes [[142], [143], [144]]. This was observed, for example, when transgenic mice were injected with either PSP or CBD human brain homogenate, the specific astrocytic features mentioned in Box 3 in PSP vs CBD were observed in mouse brain tissues [144], indicating that using this type of approach can be used to model neuropathological aspects of disease and be utilised to assess treatments targeting the toxic aggregation of tau or aS.

Another common challenge of tauopathy research is that is has mostly focused on AD throughout the years, and AD also bears Aβ pathology accumulation [129]. Therefore, studies on ferroptosis-related marker expression should consider that Aβ has been seen in most cases to accumulate earlier than tau in AD [129] and thus, it is postulated that most changes observed are inevitably influenced by the presence of these amyloidopathies, and not just tau. Accordingly, there is a gap in literature to bridge tauopathies and iron-related damage that must be addressed in the future research in order to seek further understanding of PSP/CBD disease pathophysiology and aetiology.

### 4.6 Emerging therapies in PSP/CBD

Similar to PD, emerging research has tried to target toxic inclusions of tau. Importantly, and as emphasised earlier, it is pivotal to do so while preserving the healthy function of the tau protein at the level of the nervous system. In 2018, an intravenous administration of tau humanised monoclonal antibody BIIB092 (formerly BMS-986168/IPN007) was tested in a randomised phase 1 clinical trial with healthy participants [145]. As N-terminal tau is thought to be involved in the toxic aggregation of tau by facilitating the transcellular spreading of inclusions seen in tauopathies; the BIIB092 antibody targeted this region specifically. It was observed that unbound N-terminal tau was suppressed in the CSF of treated patients, and this effect was dose-dependent without severe side effects. Currently, this has moved to a phase 2 clinical trial with PSP patients (NCT02460094 and NCT03068468), of which one trial was terminated early, while the other trial showed that the drug is tolerable and safe for PSP patients, but with no indications of PSP improvement [146]. This suggests that, while developments are being made, perhaps other cellular aspects must also be accounted for, such as reducing the oxidative stress that nerve cells experience in disease. Moreover, the PSP patients included scored at 20+ on the Mini-Mental State Examination (MMSE), which means that they already have moderate to severe cognitive and motor impairment, thus, much like the PD trials, it is possible that these treatments are also being administered at a stage where neurodegeneration and transcellular spread of tau have already caused irreversible damage.

Similarly, other advancements targeting specific metabolic nodes of the ferroptosis pathway have not shown a successful profile. In 2016, a multicenter, randomised, placebo-controlled, double-blind clinical trial was launched to investigate the effects of CoQ10, a ferroptosis inhibitor, in PSP [147]. Unfortunately, high doses of the drug did not improve disease symptoms nor progression of the disease. Similarly, on another interesting angle, other researchers have tried to target the glycogen synthase kinase-3 beta (GSK-3β) pathway (within ferroptosis) via which tau aggregation is thought to occur in tauopathy [148]. However, much like CoQ10, GSK-3β inhibition, while tolerable, failed to show improvements in a phase 2 clinical trial with PSP patients [149,150]. Nonetheless, it is noteworthy that treated patients showed decreased occipital lobe atrophy which may be a promising result. It is noteworthy that these trials, conducted in 2016 and 2014, over ten years ago, would perhaps not be characterised as “emerging” today. However, newer approaches have not been carried out making this review timely in opening up the avenue for novel targets in ferroptosis that could be optimally targeted to clear toxic tau and improve PSP/CBD.

## 5 Discussion and insights into the scientific challenges

Upon reviewing several layers of the ferroptosis pathway and its links with aS and tau, it is pivotal to discuss the critical challenges introduced at the start of the review, while offering insights into how we could tackle these in the scientific community.

*“Is iron dyshomeostasis driving parkinsonism progression? And how does that differ from normal ageing?”*

Iron dyshomeostasis and related events seem to be driving neurodegeneration related to ageing. However, recent literature has elucidated towards a direct role of ferroptosis on proteinopathy in parkinsonism progression [23,42,52,60,89,101,109,129]. Importantly, during ageing and age-related parkinsonism, one must focus on targeting pathways that normally deal with pathological inclusions, as well as oxidative stress which seemed to be a common denominator in many of the reviewed investigations. When it comes to normal ageing, the focus could be on targeting oxidative stress pathways which are impactful in setting off the ferroptosis cascade and can occur in the absence of protein inclusions. One specific target that stands out throughout the review is mitochondria. Not only has mitochondrial dysfunction been related to normal ageing and parkinsonisms [37,38,88,90,92,128,151], but it is also an important energy source for dopaminergic neurons (mostly affected in parkinsonism) and linked to toxic protein aggregates [152].

To further investigate the role of mitochondria in ferroptosis and proteinopathy development, we can use models for mitochondrial dysfunction in a parkinsonism context. There are known PD-related mutations highly linked to altered mitochondrial bioenergetics and function. PINK1 is a regulator of mitophagy, while Parkin associates with damaged mitochondria to promote its turnover [152]. Similarly, protein deglycase (DJ-1) helps to reduce mitochondrial oxidative stress and promote its bioenergetics and health [152]. Mutations to these three genes (PINK1, Parkin and DJ-1) have been well characterised in PD and linked to aS aggregation, therefore, when these genes are mutated, mitochondria are highly vulnerable and aggregation of misfolded aS is likely potentiated [152]. Additionally, F-box protein 7 (FBX07) is less characterised neuropathologically but known to have a protective function via controlled mitophagy [153]. However, alterations to this gene and protein expression have been associated with aS aggregation in PD, and with tau inclusions in PSP patients [154]. While much less is known about mutations in mitochondria-related genes in primary tauopathies, this research could be used towards developing an in vitro model by inducing mutations in the PINK1/Parkin/DJ-1/FBX07 genes in cell lines, such as the Lund human mesencephalic (LUHMES) cells. These cells are differentiated into dopaminergic neurons and allow for testing mutation-induced mitochondrial dysfunction associated with parkinsonism and then assess their vulnerability to oxidative stress when adding tau and aS fibrils in vitro. In sum, rare genetic mutations associated with parkinsonism could be used to model mitochondrial stress and dysfunction in neuronal cell lines and help understand proteinopathy and mitophagy-induced ferroptosis vulnerability in parkinsonism.

Moreover, and as mentioned, both tau and aS have crucial roles at the level of axons and synapse, respectively. Thus, moving forward with preventing parkinsonism, it is pivotal that these normal functions are preserved and potentiated by decreasing toxic proteins, and in turn also enriching their “healthy” function. Overall, the involvement of proteinopathy and ferroptosis during the progression of parkinsonism, and the potential of combined targeting could present promising results in rescuing cellular functions by clearing pathology and decreasing ferroptosis.

*“Are changes in the ferroptotic pathway a cause or consequence of pathology? Or independent of one another?”*

This question still remains an intense debate in literature (see “Graphical Abstract”). As reviewed, there are links that point to a dependency between toxic protein inclusions and increased ferroptosis. However, it is also known that normal ageing leads to changes in the ferroptotic pathway, which may differ from that seen in parkinsonisms. Moreover, most of the research has been conducted in an “overall disease model” (i.e. human, mouse, any tissue/animal with the overall disease), but findings specific to the interactions of the protein that characterises the disease at a molecular level and ferroptosis-related markers are only now emerging. Ayton et al. (2020) [139] proposed that iron de-regulation may be a parallel event to neurodegeneration that can speed up the neurodegeneration process, but without necessarily interacting with the proteinopathy directly. While all these hypotheses are worth considering, it is clear that not enough evidence is available to make a strong case against what is cause and what is consequence – but this opens the door to future research for focusing on dismantling the relationship between toxic protein inclusions and ferroptosis in the understanding of parkinsonism pathophysiology.

Can we see if proteinopathy still occurs when we inhibit ferroptosis? Or vice versa? It is known that chaperones are important components in the processes that deal with protein sorting and degradation. To further understand the research in this direction, model systems with chaperones that bind aS or tau, in the presence of exogenous fibrils of aS/tau and ferroptosis inducers or inhibitors are necessary. This could help shed some light on the interaction of ferroptosis-related markers and whether these processes affect the role of the chaperones in assisting with protein (tau/aS) misfolding.

*“What is the relationship between tau and ferroptosis in PSP/CBD? And how does it compare to aS in PD?”*

It is evident that although the normal functions of tau and aS differ, toxic aggregates of either tau and aS are detrimental to cellular functions and could lead to neurodegeneration, namely parkinsonisms. PD and PSP/CBD parkinsonisms seem to all have not only a toxic-gain-of-function but also the loss of the physiological function of aS and tau when they are not misfolded, so both angles are worth considering. As described, interactions between tau or aS with ferroptosis are distinct and not all ferroptosis-related markers have been investigated in both contexts – so the question still remains on how they could possibly compare. It is noteworthy that PD, as well as PSP/CBD are characterised by the presence of these inclusions in dopaminergic neurons and the consequent loss of these cells. The specific loss of dopaminergic neurons has a direct impact on the motor system and hence why parkinsonisms are phenotypically characterised by motor-related deficits. So why are dopaminergic neurons affected so intensely compared to other cells? This is because dopaminergic neurons are energetically demanding, compared to other nerve cells, and this energy is primarily obtained from mitochondria [152]. As dopaminergic neurons are the primary cells affected by pathology, as well as in need for higher energy amounts from mitochondria, it is tempting to speculate that, as mitochondria is central to ferroptosis, in all three parkinsonisms, the protein inclusion and consequent dopaminergic cell death may be tightly linked to mitochondrial dysfunction [152]. Mitochondrial dysfunction and oxidative stress produced, are also common hallmarks of ferroptosis but also commonly observed in parkinsonism patients’ tissues. This could be the key in connecting tau/aS to ferroptosis in parkinsonism pathophysiology. In conclusion, although tau and aS proteins bear different functions, their toxic forms preferentially affect dopaminergic neurons and consequently lead to similar clinical pictures, as well as changes in the ferroptotic pathway. Thus, focusing on mitochondria within ferroptosis may be key in preserving these dopaminergic neurons in parkinsonisms.

## 6 Conclusion

Several ferroptosis-related markers have been explored in the context of aS and tau. However, while aS research seems more advanced, most of the tau research is limited by models of AD with Aβ pathology. Therefore, many ferroptosis-related markers’ expression remains unclear in relation to tau, since the presence of Aβ is likely affecting expression and function of these proteins in these AD models, perhaps more than tau, seen as tauopathies are secondary to Aβ deposition in AD [133]. Of particular interest, and as discussed above, mitochondrial function seems to be a key element in regulating both toxic tau/aS aggregation, as well ferroptosis, but different tau isoforms also affect mitochondrial function differently [155]. This creates a gap in literature regarding what is known about ferroptosis in PSP/CBD, and how it differs from PD. Here we reviewed the current research on ferroptosis and its potential relation with tau and aS, but much of it remains unknown, especially given the controversies on what is thought to emerge first: *proteinopathy* or *ferroptosis*? Which is *cause* and which is *consequence*?

One important factor evident throughout the review is that in either aS or tau-related research, reports involve a mixture of studies with different stages of pathology and protein aggregation. For example, some of the PD studies looked into aS oligomerisation at earlier stages [39], while others also included LB pathology in their analyses [83]. Similarly, in PSP/CBD, some researchers focused on (less) phosphorylated tau [28], while others comprised more toxic hyperphosphorylated tau/NFTs [53,139]. Although it is known that increased oligomerisation/phosphorylation of proteins correlates with advanced disease progression, worsened phenotypes, and likely overall more ferroptosis, it is unclear how ferroptosis-related markers/events in studied models would behave in more (or less) advanced pathological conditions. Indeed, level of protein aggregation could also serve as a good temporal marker to help understand if the expression of ferroptosis markers would favour a more compensatory mechanism likely happening in the early disease stages, or towards a ferroptotic cell death endpoint, characteristic of later disease stages.

Furthermore, in the majority of the studies on post-mortem neurons, cell lines and/or animal models, ultimately, all measurements/analyses were performed on either surviving neurons (from post-mortem tissue) or live cells, meaning that results reported might depict a positive bias towards viable and/or resilient neurons/cells. Thus, in ferroptosis research we must consider that our marker measurements in “intact” cells are subjective to disease stage *preceding* ferroptosis, and not actually experiencing ferroptotic cell death. Studying resilience of surviving neurons is also an interesting approach to explore what may be present and causing protection, and what often creates a shift from compensatory mechanisms to cellular death.

Finally, amongst many scientific challenges here presented, and as mentioned earlier, due to the large number of misdiagnoses in parkinsonisms, it is important that we also consider how the effects of ferroptosis on tau biology compare with aS in distinct diseases, and whether ferroptosis-related markers could potentially be used as parkinsonism biomarkers to improve diagnostic tools and treatments. Similarly, it is pivotal to address how the ferroptosis pathway may change with normal ageing and if there is a shift once proteinopathies start to accumulate into age-related neurodegeneration. Interestingly, and also proposed in this review, neuromelanin, which is also lost in normal ageing, may serve as a promising avenue of research due to its iron chelating effects and thus, neuroprotective properties. In conclusion, there are still several unanswered questions in the field of ferroptosis and proteinopathies, therefore, additional research into their interplay is encouraged to close the gap in our understanding of parkinsonism pathophysiology.

## Limitations and considerations

• Cell density reports in post-mortem samples are encouraged to understand how the expression of a marker changes in relation to age-matched controls.
• Post-mortem tissues are only a “static picture in time” limiting conclusions/extrapolation from a stationary model.
• Cell lines and animal models allow observation of dynamic changes in response to treatments but translation into the clinic is often difficult.

## Next steps in research

We should be seeking out to understand two im portant factors in the understanding of ferroptosis pathway in parkinsonism:

• What comes first? Proteinopathy *or* iron dyshomeostasis (and uncontrolled ferroptosis)?
• How do different aS/tau oligomerisation stages affect ferroptosis-related marker expression?

It is also pivotal to employ novel transcriptomic techniques to explore ferroptosis-related pathways at a molecular level with inclusions and without inclusions, to understand the role of aS/tau in driving ferroptosis changes at the genome level, and to bridge the knowledge gaps in the function of specific markers in order to deepen our understanding of parkinsonism pathophysiology.

## Funding

MJdCC received a fellowship from the Behavioural and Cogntive Neurosciences Research School, awarded by the University Medical Centre Groningen in partnership with the University of Groningen. WdD is the recipient of a Parkinson Fonds grant (no. 2023\1896) and a Stitching Woelse Waard grant (Project title ‘Inhibiting ferroptosis in Parkinson's disease and variants PSP and CBD’). AMD is a recipient of a Parkinson Fonds grant (2022/1889), ZonMw Open Competitie grant. AMD was supported by a Rosalind Franklin Fellowship co-funded by the 10.13039/501100000780European Union and the 10.13039/501100001721University of Groningen.

## CRediT authorship contribution statement

**Maria João da Costa Caiado:** Writing – review & editing, Writing – original draft, Funding acquisition, Conceptualization. **Amalia M. Dolga:** Writing – review & editing, Supervision, Funding acquisition, Conceptualization. **Wilfred F.A. den Dunnen:** Writing – review & editing, Supervision, Conceptualization, Funding acquisition.

## Declaration of competing interest

The authors declare the following financial interests/personal relationships which may be considered as potential competing interests: Wilfred den Dunnen reports financial support was provided by Parkinson Fonds. Wilfred den Dunnen reports financial support was provided by Stichting Wolese Waard. Amalia Dolga reports financial support was provided by Parkinson Fonds. Amalia Dolga reports financial support was provided by ZonMw Open Competitie. Amalia Dolga reports financial support was provided by Rosalind Franklin Fellowship. Amalia Dolga reports a relationship with Parkinson Fonds that includes: board membership. If there are other authors, they declare that they have no known competing financial interests or personal relationships that could have appeared to influence the work reported in this paper.

## Data Availability

No data was used for the research described in the article.
